# Exploring the genetic basis of gene transcript abundance and metabolite levels in loblolly pine (*Pinus taeda* L.) using association mapping and network construction

**DOI:** 10.1186/s12863-018-0687-7

**Published:** 2018-11-06

**Authors:** Mengmeng Lu, Candace M. Seeve, Carol A. Loopstra, Konstantin V. Krutovsky

**Affiliations:** 1 Department of Ecosystem Science and Management, Texas A&M University, 2138 TAMU, College Station, TX 77843-2138 USA; 2 Molecular and Environmental Plant Sciences Program, Texas A&M University, 2474 TAMU, College Station, TX 77843-2474 USA; 3 Department of Biological Sciences, University of Calgary, 507 Campus Drive NW, Calgary, AB T2N 4S8 Canada; 4 USDA-ARS Midwest Area, Columbia, MO 65211 USA; 5 Department of Forest Genetics and Forest Tree Breeding, Georg-August University of Göttingen, Büsgenweg 2, 37077 Göttingen, Germany; 6 Laboratory of Population Genetics, Vavilov Institute of General Genetics, Russian Academy of Sciences, Gubkina Str. 3, Moscow, 119333 Russia; 7 Laboratory of Forest Genomics, Genome Research and Education Center, Siberian Federal University, 50a/2 Akademgorodok, Krasnoyarsk, 660036 Russia

**Keywords:** Gene expression, Metabolism, Epistasis, Stress response, Wood development, SNP

## Abstract

**Background:**

Identifying genetic variations that shape important complex traits is fundamental to the genetic improvement of important forest tree species, such as loblolly pine (*Pinus taeda* L.), which is one of the most commonly planted forest tree species in the southern U.S. Gene transcripts and metabolites are important regulatory intermediates that link genetic variations to higher-order complex traits such as wood development and drought response. A few prior studies have associated intermediate phenotypes including mRNA expression and metabolite levels with a limited number of molecular markers, but the identification of genetic variations that regulate intermediate phenotypes needs further investigation.

**Results:**

We identified 1841 single nucleotide polymorphisms (SNPs) associated with 191 gene expression mRNA phenotypes and 524 SNPs associated with 53 metabolite level phenotypes using 2.8 million exome-derived SNPs. The identified SNPs reside in genes with a wide variety of functions. We further integrated the identified SNPs and the associated expressed genes and metabolites into networks. We described the SNP-SNP interactions that significantly impacted the gene transcript abundance and metabolite level in the networks. Key loci and genes in the wood development and drought response networks were identified and analyzed.

**Conclusions:**

This work provides new candidate genes for research on the genetic basis of gene expression and metabolism linked to wood development and drought response in loblolly pine and highlights the efficiency of using association-mapping-based networks to discover candidate genes with important roles in complex biological processes.

**Electronic supplementary material:**

The online version of this article (10.1186/s12863-018-0687-7) contains supplementary material, which is available to authorized users.

## Background

Understanding the genetic basis of complex phenotypes in the important forest tree species loblolly pine (*Pinus taeda* L.) can contribute to the improvement of its growth and quality. Genetic variation does not lead to changes in whole-plant traits directly, but instead acts through intermediate, molecular phenotypes, which in turn induce changes in higher-order traits [1]. Gene transcripts and metabolites are measurable intermediates that link genetic variations to whole-plant phenotypes. They are regulated by genetic and environmental cues, and perturbations in these intermediate phenotypes can directly or interactively affect higher-order traits [1]. Thus, studies linking gene expression or metabolite phenotypes to genetic variations may enhance our understanding of the molecular mechanisms that underlie broader whole-plant phenotypes. For example, Bossu et al. [2] found secondary metabolites influence wood properties. Obata et al. [3] demonstrated that metabolite levels in maize respond to stress conditions and can be used to predict the grain yield under drought.

The majority of previous genetic studies on loblolly pine have focused on the dissection of adaptive or commercially important traits, including growth, wood properties, or drought tolerance [4–7], while only a few studies have sought to associate intermediate phenotypes, such as levels of transcripts and metabolites with genome-wide genetic variation. The number of molecular markers used in these studies was limited to 3000–4000 SNPs [8–10]. Palle et al. [8] analyzed expression of genes involved in loblolly pine wood development, and detected associations between mRNA expression level of 33 wood development genes (expression phenotypes) and 80 single nucleotide polymorphisms (SNPs). Seeve [9] measured mRNA levels of 88 genes related to loblolly pine disease or drought responses in loblolly pine and found that 27 expression phenotypes were associated with 94 SNPs. Eckert et al. [10] detected 28 SNP-metabolite associations in loblolly pine. These seminal studies identified candidate genes and alleles associated with gene expression and metabolite phenotypes, but the limited number of molecular markers used in these studies constrain our understanding of the genetic basis underlying these complex intermediate phenotypes.

Determining how the identified gene candidates from association mapping analyses are organized to function in complex biological processes is a difficult problem that needs to be addressed. One of possible methods to address this problem is to integrate SNPs and their associated gene expression and metabolite level phenotypes into networks. In doing so, we can understand developmental and stress resistance phenotypes in a system response rather than as a product of individual genes [11]. In addition, we can identify key genes in networks that contribute to adaptive traits [12, 13]. However, this network construction method has not been previously used to examine gene relationships in loblolly pine.

In the present study, we tested for associations between 2.8 million SNPs derived from exome target sequencing [14, 15] and intermediate, molecular phenotypes measured as gene transcript [8, 9] and metabolite [10] levels. We further constructed gene networks to analyze the loci associated with multiple phenotypes. Since epistatic interaction between loci is another factor that may further influence phenotypes in loblolly pine [15], SNP-SNP interactions were also studied among the identified loci. The large number of SNPs used in this study enabled the discovery of novel loci that are associated with intermediate phenotypes linked to the agronomically important higher-order traits — disease resistance, drought tolerance, and wood development. The networks also enable us to identify key gene candidates to study further the genetic basis of regulatory and biosynthetic pathways in loblolly pine.

## Results

### Significant associations between SNPs and phenotypes

**Fig. 1:**
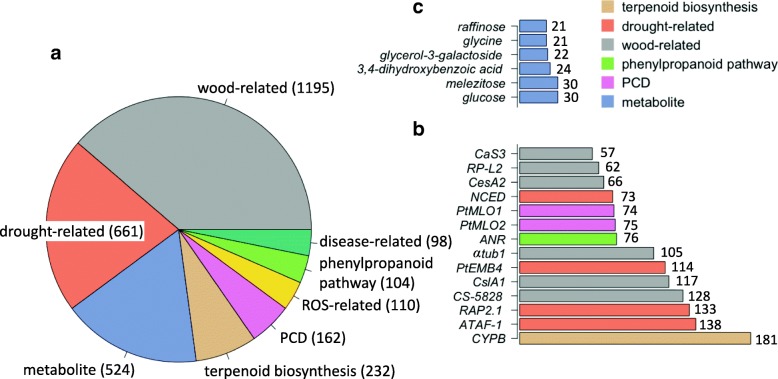
Number of significant SNP-phenotype associations for (**a**) metabolites and 7 different functional groups of genes, for which expression level was used as a phenotypic trait in the SNP association study, (**b**) 14 genes and (**c**) 6 metabolites with the most SNP associations

We identified a total of 2562 associations between 1841 SNPs and 191 gene expression phenotypes and 524 associations between 524 SNPs and 53 metabolite concentration phenotypes (Fig. 1a, Additional file 1: Tables S1 and S2). A total of 40% and 23% of the SNPs associated with gene expression and metabolite concentration phenotypes, respectively, had a minor allele frequency (MAF) ≥ 0.05. The MAFs of other SNPs were between 0.01 and 0.05. Genotypes of 9% of the SNPs associated with gene expression and 6% of the SNPs associated with metabolite concentrations were not in Hardy-Weinberg equilibrium. Among the 2562 gene expression associations (Fig. 1a), 1195 were related to expression of wood development genes, 661 to drought-related genes, and others to programmed cell death (PCD) genes, reactive oxygen species (ROS) genes, phenylpropanoid pathway genes and disease-related genes. Expression of the *CYPB* gene (involved in terpenoid biosynthesis) was associated with the largest number of SNPs (181 SNPs). It was followed by genes encoding a drought-responsive transcription factor (TF) *ATAF-1* (138 SNPs), a drought-responsive TF *RAP2.1* (133 SNPs) and other genes (Fig. 1b, Additional file 1: Tables S3). Levels of the metabolites glucose and melezitose were each associated with 30 SNPs (Fig. 1c, Additional file 1: Tables S4).

We found that *r*^2^ values for the SNP-trait associations showed a distinct difference between SNP-gene-expression and SNP-metabolite-level associations. Although the median of *r*^2^ values was 0.15 for both groups, the *r*^2^ values of SNP-gene-expression associations had a wide range, from 0.09 to 0.85, while the *r*^2^ values of SNP-metabolite-level associations ranged from 0.11 to 0.22 (Additional file 2: Figure S1). We examined the 323 SNP-gene expression associations with high *r*^2^ values (> 0.40). Among them 181 were associated with the *CYPB* gene, 133 with the *RAP2.1* gene, four with the *PtMLO1* gene involved in PCD, two with the peroxidase *PtGPX3* gene, two with the cellulose synthase *CesA2* gene, and one with the callose synthase *CaS3* gene.

We found that the SNPs scaffold596656_40783 and tscaffold2197_12732 discovered in the current study reside in genes also identified in the prior study [16]. The SNP scaffold596656_40783 was associated with expression of the *CAD1* gene encoding cinnamyl-alcohol dehydrogenase involved in a lignin biosynthesis. This SNP resides in a gene encoding cystathionine gamma-synthase. The SNP tscaffold2197_12732 was associated with expression of the *CesA2* gene. This SNP resides in a gene encoding E3 ubiquitin-protein ligase. Other identified SNPs in this study could not be mapped to the genes identified in prior studies.

### Annotation of the genes containing identified SNPs

The SNPs that were associated with gene expression phenotypes resided in 1635 different annotated genes. Among them, 57% resided in coding sequences (CDS), 2% in 5′ untranslated sequences (5’UTR), 3% in 3′ untranslated sequences (3’UTR), 23% in introns, 7% in putative 3′ regulatory sequences (P3’RS) and 8% in putative 5′ regulatory sequences (P5’RS). The SNPs that were associated with metabolite level phenotypes resided in 374 different annotated genes. Of these, 58% resided in CDS, 2% in 5’UTR, 2% in 3’UTR, 25% in introns, 6% in P3’RS, and 7% in P5’RS. The SNP-containing genes encode proteins with functions of nucleic acid binding, transporter, oxidoreductase, transferase, hydrolase, receptor, enzyme modulator, ligase, cytoskeletal protein, TF, membrane traffic protein, and signaling molecule chaperone. The major molecular functions of SNP-containing genes include catalytic activity, DNA binding, transporter activity, receptor activity and structural molecule activity.

Among the identified associations, some gene expression phenotypes were associated with a large number of SNPs. For example, expression of the *CYPB* gene that encodes a terpenoid biosynthesis enzyme, cytochrome P450 monooxygenase, was associated with 181 SNPs. The SNPs associated with *CYPB* gene expression mainly resided in genes involved in secondary metabolites biosynthesis and defense resistance, including genes encoding beta-glucosidase, phosphofructokinase, polygalacturonase, shikimate O-hydroxycinnamoyltransferase-like, cytochrome P450 78A3, glucosinolate transporter-2, TIR-NBS-LRR protein, serine/threonine protein kinase, and lipase. The expression phenotypes of genes encoding drought-responsive TFs, *RAP2.1* and *ATAF-1*, were also associated with a large number of SNPs, 133 and 138 SNPs, respectively. The associated SNPs mainly resided in drought responsive genes or TF genes that confer drought tolerance to plants including genes encoding cysteine-rich receptor-like protein, glucan endo-1,3-beta-glucosidase, COBRA-like protein, cinnamoyl-CoA reductase, root phototropism protein, putative TIR-NBS-LRR protein, laccase, cellulose synthase, UDP-glucuronyltransferase-like protein, and TFs of ethylene-responsive, bHLH, MADS-box and MYBs. Table 1 presents a partial list of the genes containing SNPs associated with gene expression and metabolite level phenotypes. More details are presented in Additional file 1: Tables S1 and S2.

**Table 1:** Functional groups for genes with expression phenotypes and genes containing SNPs associated with gene expression or metabolite level phenotypes

Functional group	Genes containing SNPs associated with gene expression or metabolite level phenotypes
Wood-related	arabinosyltransferase ARAD1; aspartokinase 3, chloroplastic-like; transcription factor GAMYB; eukaryotic translation initiation; cellulose synthase A catalytic subunit; clathrin assembly protein, putative; vacuolar protein sorting-associated; mediator of RNA polymerase II; U3 small nucleolar RNA-associated; 1-phosphatidylinositol 3-phosphate; 60S ribosomal protein L8; condensation complex subunit 1 domain-containing; serine/threonine-protein; laccase
Drought-related	transcription factor GAMYB; oxidoreductase; heat stress transcription factor; transcription factor bHLH120-like; lactosylceramide 4-alpha-galactosyltransferase; glutamate decarboxylase 1; cytochrome b-245 beta chain homolog RbohAp108; galactomannan galactosyltransferase; UDP-glucuronyltransferase-like protein; serine carboxypeptidase S10 family protein; cellulose synthase A catalytic subunit 6; GDSL esterase/lipase At1g74460-like; LRR receptor-like; heat shock 22 K family protein; bidirectional sugar transporter; myrosinase-binding protein-like protein; myo-inositol-1-phosphate synthase 2
Disease-related	serine carboxypeptidase-like; LRR receptor-like; serine/threonine-protein; lipoxygenase
Phenylpropanoid pathway	brassinosteroid-regulated protein; calcium-dependent protein kinase 3-like
Reactive oxygen species (ROS)-related	MATE efflux family protein; polyadenylate-binding protein; cytochrome b245 beta chain homolog RbohAp108; glutathione peroxidase
Terpenoid biosynthesis	(−)-alpha-terpineol synthase; MADS-box transcription factor 6-like
Programmed cell death (PCD)	cytochrome P450; MLO-like protein 12; putative NBS-LRR protein; late embryogenesis abundant protein LEA8–4; MADS-box transcription factor 6-like; ethylene-responsive transcription factor
Metabolite-related	cytochrome P450; peroxidase; leucine-rich repeat transmembrane protein kinase; probable serine/threonine-protein; cysteine-rich receptor-like protein; laccase; WD repeat-containing protein; transcription factor MYB108-like; probable E3 ubiquitin-protein ligase; AP2/ERF domain-containing transcription factor; cellulose synthase-like protein D2; cyclin-dependent kinase B1–1; ent-copalyl diphosphate synthase; glutathione S-transferase DHAR3; 4-coumarate--CoA ligase-like 5; bifunctional UDP-glucose 4-epimerase; TMV resistance protein N-like; UDP-glycosyltransferase 85A3-like

**Fig. 2:**
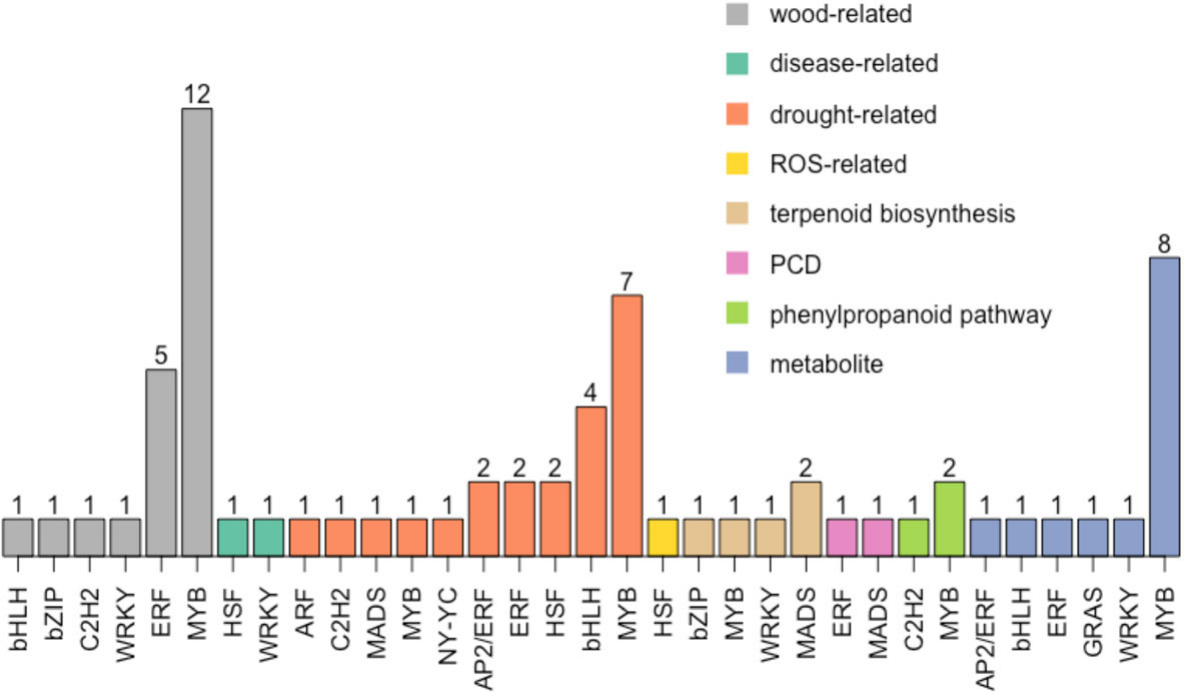
Number of significant SNP associations with gene expression and metabolite concentration level phenotypes for 59 transcription factor (TF) genes representing 12 TF families AP2/ERF, ARF, bHLH, bZIP, C2H2, ERF, GRAS, HSF, MADS, MYB, NY-YC, and WRKY with 69 SNPs associations, in total. The gene with expression phenotypes were classified into seven different functional groups: wood-related, disease-related, drought-related, reactive oxygen species (ROS)-related, terpenoid biosynthesis, programmed cell death (PCD), and phenylpropanoid pathway. The numbers above each bar represent the numbers of identified SNPs associated with gene expression or metabolite level phenotypes

We found 59 genes representing 12 TF families and containing 69 SNPs significantly associated with gene expression or metabolite level (Fig. 2). Among them, 26 genes belong to the MYB family and contain 31 SNPs associated with expressed genes encoding a wood development protein (*1CAB-3A*), cellulose synthase (*CesA)*, a cell wall protein (*CslA*), α-tubulin (*αtub1*), a lignin biosynthesis enzyme (*TC4H*), a drought-responsive TF (*RAP2.1*), a phenylpropanoid pathway enzyme (*ANR*) and also with metabolites 4-hydroxybenzoate, aspartic acid, cellobiose, coniferin, glutamic acid, glycerol-3-galactoside, maltose and melezitose. Details of the TFs annotations, SNPs and their associated phenotypes are presented in Additional file 1: Table S5.

### Linkage disequilibrium (LD) among identified SNPs that resided in the same scaffolds

**Fig. 3:**
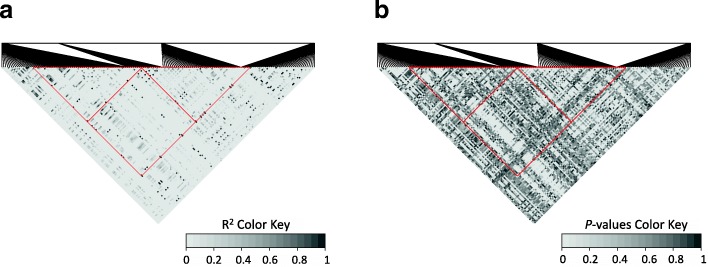
Pairwise linkage disequilibrium (LD) plots for SNPs in the scaffold tscaffold2867 with *R*^2^ (**a**) and their *P*-values (**b**) depicted by different black and white color shades. The bottom vertex of each red triangle outlines the significant LD values for SNPs tscaffold2867_628232, tscaffold2867_651263 and tscaffold2867_755157 (*R*^2^ > 0.89, *P* < 0.01)

We identified 10 scaffolds containing SNP pairs with significant LD, but did not observe long stretching LD blocks (Fig. 3, Additional file 2: Figures S2-S10). In some cases, loci that are more than 10 Kbp apart along the same scaffolds were in LD and associated with the same gene expression phenotypes with similar *r*^2^ values. For example, the SNPs tscaffold2867_628232, tscaffold2867_651263, and tscaffold2867_755157 span 128 Kbp on tscaffold2867, but all three were associated with expression of the *ATAF-1* gene (drought-responsive TF) with *r*^2^ = 0.31. We detected high pairwise LD values (> 0.89) between these SNPs. However, we did not observe long LD blocks along the investigated regions in the LD heatmaps (Fig. 3, Additional file 2: Figures S2-S10).

### Gene networks

**Fig. 4:**
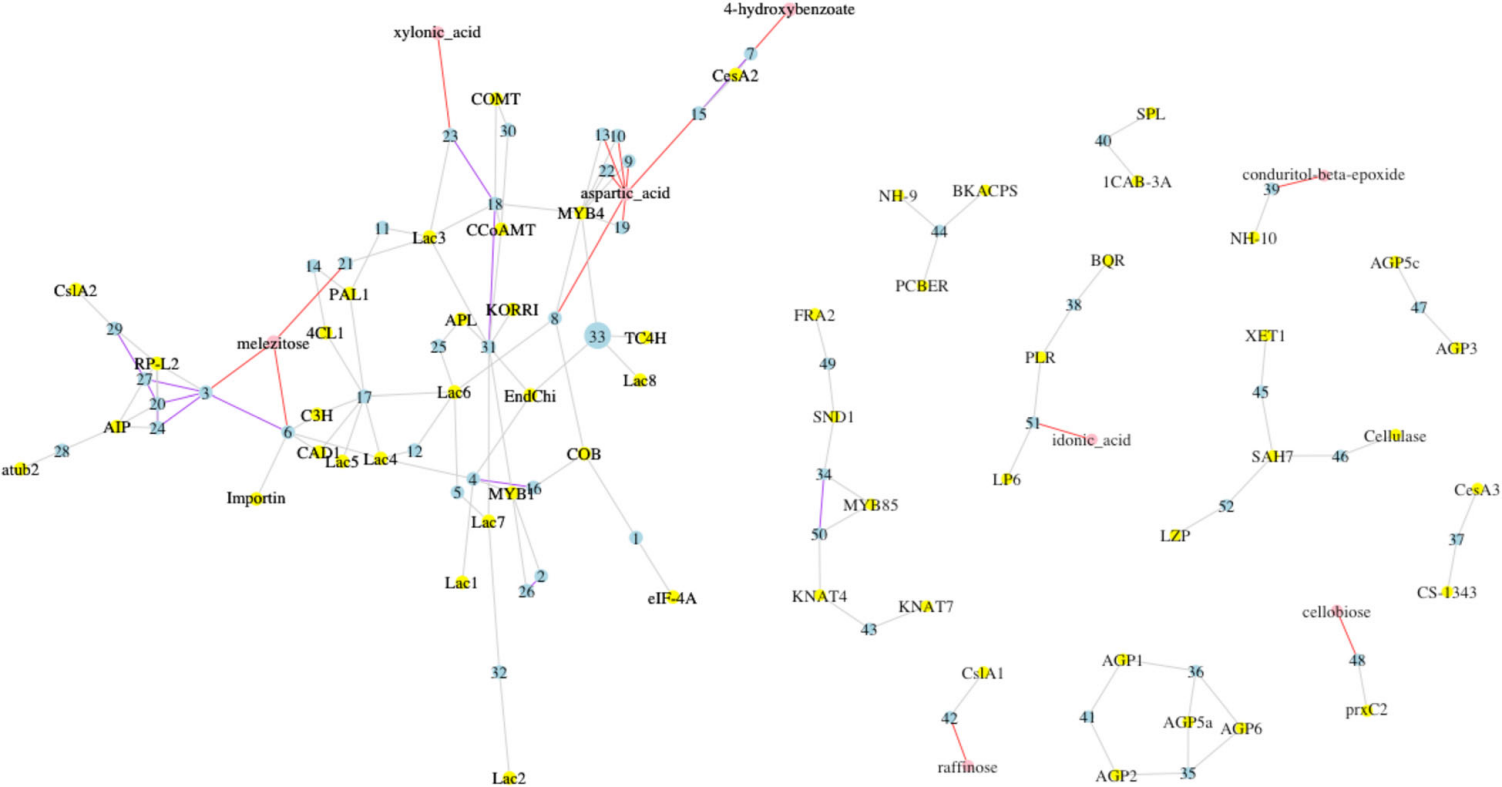
Gene networks comprised of SNPs and their associated wood-related gene expression and metabolite level phenotypes. The blue dot nodes represent SNPs. Details of the SNPs and the genes containing them are presented in Table 2. The large blue dot node 33 represents a SNP that resides in a GAMYB transcription factor (TF) gene. The yellow dot nodes represent genes, for which expression level was used as a phenotype trait in the SNP association analysis. The pink dot nodes represent metabolites, for which concentration level was used as a phenotype trait in the SNP association analysis. The grey and red edges represent SNP-gene-expression and SNP-metabolite-level associations, respectively. The purple edges represent SNP-SNP interactions that significantly impact the phenotypes. Expressed genes in the network include arabinogalactan-protein and cell wall protein genes (*AGP1–6*), cell expansion genes (*COB* and *KORRI*), cell wall related (resistance related) genes (*CslA1*), cellulose and callose synthase genes (*CesA3*, *CslA2*, and *CS-1343*), lignin biosynthesis enzyme genes (*4CL1*, *C3H*, *CAD1*, *CCoAMT*, *COMT*, *Lac1–8*, *PAL1*, and *TC4H*), α-tubulin gene (*αtub2*), wood development enzyme genes (*BKACPS*, *BQR*, *Cellulase*, *EndChi*, *Importin*, *LP6*, *PCBER*, *PLR*, *prxC2*, *SAH7*, *SPL*, and *XET1*), wood development protein genes (*1CAB*-3A, *NH-10*, *NH-9*, and *RP-L2*), wood development TF genes (*SND1*, *AIP*, *APL*, *eIF-4A*, *FRA2*, *KNAT4*, *KNAT7*, *LZP*, *MYB1*, *MYB4*, and *MYB85*)

**Fig. 5:**
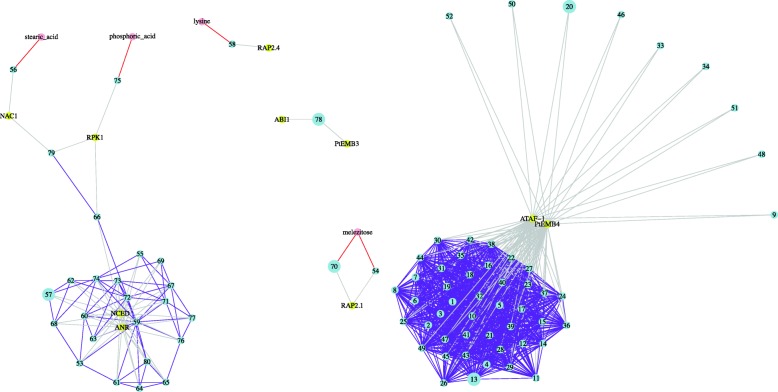
Gene networks comprised of SNPs and their associated drought-related gene expression and metabolite level phenotypes. Blue dot nodes represent SNPs. Details of the SNPs and the genes containing them are presented in Table 3. The blue dot nodes 13, 20, 57, 70, and 78 with a larger size represent the SNPs that reside in transcription factor (TF) genes. The yellow dot nodes represent genes, for which expression level was used as a phenotype trait in the SNP association analysis. The pink dot nodes represent metabolites, for which concentration level was used as a phenotype trait in the SNP association analysis. The grey and red edges represent SNP-gene-expression and SNP-metabolite-level associations, respectively. The purple edges represent SNP-SNP interactions that significantly impact the phenotypes. Expressed genes in the network include drought signaling genes (*ABI1*, *NCED*, and *RPK1*), drought-responsive TF genes (*NAC1*, *RAP2.1*, *RAP2.4*, and *ATAF-1*), late embryogenesis abundant protein genes (*PtEMB3*–4), phenylpropanoid pathway gene (*ANR*)

The wood development (Fig. 4) and drought response (Fig. 5) gene networks contained the largest number of SNPs (Tables 2 and 3 and Additional file 2: Tables S7 and S8). In the wood development gene network, 52 SNPs (each represented as a number in a blue dot node in Fig. 4) were associated with 56 gene expression phenotypes (represented by yellow dot nodes and grey edges in Fig. 4) and 8 metabolite level phenotypes (represented by pink dot nodes and red edges in Fig. 4). In the drought response gene network, 80 SNPs (each represented as a number in a blue dot node in Fig. 5) were associated with 10 gene expression phenotypes (represented by yellow dot nodes and grey edges in Fig. 5) and 4 metabolite level phenotypes (represented by pink dot nodes and red edges in Fig. 5). SNP #33 in the wood development network (Fig. 4) and SNPs #13, #20, #57, #70 and #78 in the drought response network reside in TF genes (Fig. 5).

**Table 2:** Annotation of genes containing SNPs associated with gene expression or metabolite level related to wood development

SNP#	Gene function	SNP#	Gene function
1	^a^BTB-POZ and MATH domain 6	27	pentatricopeptide repeat-containing
2	heat shock cognate 71 kDa protein	28	sugar transport protein 7-like
3	^a^dormancy/auxin associated family	29	uninformative
4	^a^lipoxygenase LOX2	30	E3 ubiquitin-protein ligase RHA1B-like
5	transmembrance protein, UPF0481	31	aspartokinase 3, chloroplastic-like
6	clathrin assembly protein, putative	32	uninformative
7	laccase 5	33	transcription factor GAMYB
8	1-phosphatidylinositol 3-phosphate	34	putative peptide/nitrate transporter
9	pleiotropic drug resistance protein	35	eukaryotic translation initiation
10	pleiotropic drug resistance protein	36	60S ribosomal protein L8
11	cytochrome P450 71A1-like	37	disease resistance family protein / LRR family
12	D-xylose-proton symporter-like 2-like	38	uninformative
13	pleiotropic drug resistance protein	39	putative peptide/nitrate transporter
14	putative chloroplast nucleoid DNA binding protein	40	D-xylose-proton symporter-like 3
15	^a^octicosapeptide/Phox/Bem1p domain	41	laccase-15-like
16	zinc finger A20 and AN1	42	L-lactate dehydrogenase A isoform X2
17	probable arabinosyltransferase ARAD1	43	chalcone synthase
18	^a^disease resistance protein	44	serine/threonine-protein
19	^a^wall associated kinase 3	45	not_annotated
20	^a^U1 snRNP 70 K truncated protein	46	GTP pyrophosphokinase-like
21	calcium-dependent protein kinase	47	beta-galactosidase 8-like
22	pleiotropic drug resistance protein	48	zinc finger protein, putative
23	probable LRR receptor-like	49	glutaredoxin-C1
24	histone H2A 11	50	tir-nbs-lrr resistance protein
25	CBL-interacting protein kinase 04	51	tubulin-folding cofactor D-like
26	^a^cytochrome P450 CYP866A1	52	early endosome antigen 1

^a^Based on the NCBI blastx results

**Table 3:** Annotation of genes containing SNPs associated with gene expression or metabolite level related to drought response

SNP#	Gene function	SNP#	Gene function
1	^a^uninformative	41	phospholipase D beta 1-like
2	^a^pentatricopeptide repeat-containing protein	42	DNA helicase INO80-like
3	^a^PQ-loop repeat family protein	43	kinesin-4-like
4	^a^protein sensitivity to red light reduced 1	44	^a^NBS/LRR, partial
5	wall-associated receptor kinase-like 14	45	LRR receptor-like
6	probable mediator of RNA polymerase II	46	^a^uninformative
7	myrosinase-binding protein-like protein	47	L-ascorbate oxidase homolog
8	PHD finger protein alfin-like 5 isoform	48	MLO protein homolog 1-like
9	GDSL esterase/lipase	49	prephenate dehydrogenase family protein
10	guanine nucleotide-binding protein-like	50	^a^uninformative
11	^a^uninformative	51	UDP-glucuronyltransferase-like protein
12	bidirectional sugar transporter	52	protein transparent testa 12-like
13	heat stress transcription factor	53	^a^Cytochrome P450 CYP736B9
14	^a^F6 N18.1	54	^a^Cytochrome P450 CYP736B9
15	^a^leukocyte immunoglobulin-like receptor familyA	55	^a^nucleotide-diphospho-sugar transferases
16	pentatricopeptide repeat-containing	56	cytochrome P450 71A1-like
17	cellulose synthase A catalytic subunit 6	57	transcription factor HY5-like
18	probable acyl-activating enzyme 1	58	expansin-B3 isoform X2
19	cytochrome b245 beta chain homolog RbohAp108	59	^a^transmembrane protein (DUF616)
20	transcription factor bHLH120-like	60	peroxygenase 2
21	heat shock 22 K family protein	61	14–3-3-like protein-like
22	^a^phosphatidylethanolamine-binding protein	62	L-type lectin-domain containing receptor kinase
23	^a^villin 2, actin binding protein	63	L-type lectin-domain containing
24	DNA topoisomerase 2	64	^a^probable nucleoredoxin 1
25	^a^copia-like polyprotein	65	^a^sulfite oxidase
26	^a^membrance trafficking family	66	thioesterase/thiol ester dehydrase-isomerase
27	lactosylceramide 4-alpha-galactosyltransferase	67	uninformative
28	galactomannan galactosyltransferase	68	chaperone protein ClpB1-like
29	pleiotropic drug resistance protein	69	60S ribosomal protein L7a
30	actin	70	^a^myb domain protein 17
31	^a^CML42, calcium-binding protein	71	homeobox-leucine zipper protein
32	^a^SWEET1, bidirectional sugar transporter	72	probable pectinesterase 53-like,
33	uninformative	73	^a^SUS2, sucrose synthase 2
34	GDSL esterase/lipase At1g74460-like	74	^a^caleosin-related family protein
35	^a^glycosyltransferase,partial	75	transaldolase 2
36	oxidoreductase	76	calcium-dependent protein kinase 3-like
37	^a^armadillo/beta-catenin repeat family protein	77	calcium-dependent protein kinase 3-like
38	^a^sedoheptulose-bisphosphatase	78	transcription factor GAMYB
39	^a^endonuclease/exonuclease/phosphatase family	79	UPL2, similar to E3 ubiquitin protein ligase
40	not_annotated	80	^a^uninformative

^a^Based on NCBI blastx results

We also identified a few associations between SNPs and expression phenotypes of genes belonging to other functional groups included in this study. For instance, a limited number of connections were found in the reactive oxygen species (ROS) response and disease response gene networks (presented in Additional file 2: Figure S11 and Table S9). No networks were found for expression phenotypes of genes related to terpenoid biosynthesis, PCD or the phenylpropanoid pathway.

We identified several large gene networks that are thought to contribute significantly to two whole-plant phenotypes examined in this study - wood development and drought response, respectively. Figure 4 presents several small and one large gene networks related to wood development. The large network contains 33 SNPs, 4 metabolites and 28 expressed genes that encode cellulose and callose synthases, lignin biosynthetic enzymes, wood development enzymes, and tubulins. Figure 5 presents three small and two large gene networks related to drought response processes. One large network is composed of 24 SNPs, 2 metabolites and 4 expressed genes that encode drought response TFs, drought signaling molecules and phenylpropanoid pathway enzymes. The other large network contained 52 SNPs and two expressed genes that encode a drought responsive TF and a late embryogenesis abundant protein.

## Discussion

In this study, we identified 1841 SNPs associated with 191 gene expression phenotypes and 524 SNPs associated with 53 metabolite level phenotypes. These identified SNPs provide valuable resources to help with our understanding of the regulation of gene expression and metabolism related to wood development and stress response. We constructed gene networks to present the potential interactions among loci and to prioritize the candidate genes that are linked to biosynthesis and regulation of wood development and drought response through molecular intermediates. These results provide valuable data to bridge connections between genetic variation, phenotypes emerging at intermediate levels of biological organization, and whole-plant phenotypes.

We detected an array of SNPs significantly associated with gene expression phenotypes with a wide range of *r*^2^values (the proportion of phenotypic variation that is explained by the corresponding markers) from 0.09 to 0.85. However, associations were less strong for SNP-metabolite level associations (*r*^2^ values ranged from 0.11 to 0.22), possibly because the genetic basis underlying secondary metabolism involves more complex factors.

Among the SNP-gene expression associations, we detected 181 associations with *CYPB* gene expression and 133 associations with *RAP2.1* gene expression that had remarkably high *r*^2^ values, ranging from 0.40 to 0.85 indicating that the associated markers can explain a large proportion of the variation in mRNA level of these genes. The *CYPB* gene encodes a cytochrome P450 monooxygenase enzyme involved in the synthesis of diverse oleoresin terpenoids important for constitutive and induced defenses against pests and pathogens [17]. Correspondingly, the SNPs associated with *CYPB* gene expression were discovered in genes involved in secondary metabolite biosynthesis and defense pathways, including genes encoding a NBS-LRR type disease resistance protein and a MADS-box TF. The *RAP2.1* gene encodes a dehydration-responsive-element binding (DREB) protein type transcriptional repressor. SNPs associated with *RAP2.1* gene expression were likewise discovered in drought responsive genes or TF genes that contribute to drought tolerance, including genes encoding MYBs, which play roles in controlling responses to biotic and abiotic stresses [18]. Although the effects of genes containing the identified SNPs on the expressed genes need to be confirmed by forward genetics experiments, association studies are an efficient method to discover clusters of candidate genes in biosynthetic pathways contributing to complex traits.

We detected loci located more than 10 Kbp apart along the same scaffolds that were associated with the same gene expression phenotypes and had similar *r*^2^ values. This observation could indicate that these SNPs are in LD with each other or are even located within LD blocks. Although randomly mating outcrossing conifer trees with large effective population sizes are expected to have a rapid decline of LD, the rate of LD decay may vary from gene to gene [19–21]. Loci that both are associated with the same phenotypes and are in LD likely have strong epistatic interaction and are under selection [22, 23]. In the current study, although we detected ten scaffolds that contained identified SNPs in strong LD with each other, we did not observe long LD blocks for the regions surrounding the correlated SNPs (see heatmap plots in Fig. 3 and Additional file 2: Figures S2-S10). These results diminish the possibility that natural selection causes interactions among the investigated loci, since large blocks of LD should be maintained if the interacted loci are under selection [22, 23]. It is likely that the occasional LD observed here are artifactual and arose from mixing subpopulations with different allele frequencies [23]. The population used in this study was comprised of individuals with parents from a wide range across the southeastern U.S. Some of these artifactual LDs could be also due to potential misassembly of contigs and scaffolds.

Highly connected genes positioned within gene networks are predicted to be important “hub” genes that contribute significantly to complex traits. In the wood development gene network multiple hub genes were explored in more depth (Fig. 4). SNP #33 resides in a TF *GAMYB* gene, which has been identified as an activator of gibberellin (GA)-regulated genes in plant growth [24]. SNP #33 is associated with expressed genes encoding wood development and lignin biosynthetic enzymes, indicating that the *GAMYB* gene may influence lignin biosynthesis and wood formation through its regulatory interactions with a large number of genes. SNP #17 resides in a gene encoding arabinosyltransferase *ARAD1* that catalyzes the polymerization of arabinose into the arabinan of arabinogalactan during secondary wall formation in loblolly pine [25, 26]. SNP #17 is associated with seven gene expression phenotypes all related to lignin biosynthesis. The associations between SNP #17 and lignin biosynthesis gene expression phenotypes imply a link between arabinogalactan proteins and lignin biosynthesis for cell wall formation. SNP #31 resides in a gene encoding aspartokinase that catalyzes the phosphorylation of aspartic acid. Bacterial studies have demonstrated that decreasing aspartokinase activity results in blockage of cell wall growth [27]. SNP #31 is associated with multiple lignin biosynthesis and wood development gene expression phenotypes, suggesting aspartokinase-mediated amino acid metabolism is involved in cell wood development and lignin biosynthesis.

From the network in Fig. 4, we can identify an array of candidate genes that are associated with expression of different laccase gene family members. Laccase provides oxidative capacity during lignification. *Lac3* gene expression was associated with SNPs that reside in genes encoding a cytochrome, a disease resistance protein, a calcium dependent protein kinase, a LRR receptor-like protein and an aspartokinase. *Lac6* gene expression is associated with SNPs that reside in genes encoding a transmembrance protein, 1-phosphatidylinositol 3-phosphate, an arabinosyltransferase and a CBL-interacting protein kinase. These associations provide clues to understand the laccase oxidation process during lignification.

We gain a more complete understanding by incorporating SNP-SNP epistatic interactions into the networks. In the wood development network (Fig. 4), *RP-L2* (ribosomal protein L2) gene expression is impacted by interactions of multiple SNP-SNP pairs. *RP-L2* and 23S RNA are candidates for catalyzing peptide bond formation on the 50S subunit [28]. The SNP-SNP interactions suggest genes encoding a dormancy/auxin associated protein, pentatricopeptide repeat-containing protein and histone H2A interact to affect the formation of ribosomal proteins. Additionally, interaction between an aspartokinase gene and a disease resistance gene significantly influences *CCoAMT* gene expression, but the mechanism remains unclear. It should be noted that SNP-SNP interactions identified in the present study were general estimates because we did not take into account potential population substructure structure or kinship.

The drought response gene network highlighted four gene expression phenotypes centered in clusters of SNP associations (Fig. 5). *NCED* is a key enzyme in abscisic acid (ABA) biosynthesis, which is induced by drought stress. *ANR* functions in the phenylpropanoid pathway. Expression of the *NCED* and *ANR* genes are widely associated with the same set of SNPs, which mainly reside in genes encoding drought responsive products. This result suggests *ANR* and *NCED* genes play key roles in the drought response pathway. *PtEMB4* is a Late Embryogenesis Abundant protein. The *ATAF-1* gene belongs to the *NAC* (No Apical Meristem) family of genes, which encode plant-specific TFs involved in diverse biological processes [29]. We found expression of the *ATAF-1* and *PtEMB4* genes were associated with the same 52 SNPs, which reside in genes encoding proteins such as a wall-associated receptor kinase-like and a heat stress TF.

We found some SNPs were associated with both drought-related gene expression phenotypes and metabolite level phenotypes (Fig. 5). Since metabolic changes in response to drought conditions play a key role for drought adaptation in plants [30], the genes containing the SNPs and the expressed genes provide candidates to analyze the genetic basis of metabolic changes in response to drought. Drought stress increases stearic acid [31]. SNP #56 resides in a gene encoding a cytochrome P450. It is associated with stearic acid concentration and *NAC1* (a drought-responsive TF) gene expression. SNPs #54 and #70 are associated with melezitose concentration and *RAP2.1* gene expression. Melezitose is found in the manna of many pine trees. During droughts, bees that collect manna from these trees produce honey containing elevated concentrations of melezitose [32]. SNPs #54 and #70 reside in genes encoding a cytochrome P450 and a MYB domain protein, respectively. It is possible that biosynthesis of melezitose in response to drought is under regulation of drought responsive genes.

This study is an attempt to compose networks for exploring the genetic basis of gene expression and metabolite levels involved in complex biological processes. A total of 2.8 million SNPs were used to do association mapping, yet the numbers of investigated genes and metabolites were too limited to cover all the genes related to the biosynthetic pathways. Numbers of genes related to ROS, PCD, terpenoid biosynthesis and phenylpropanoid pathway were too few to compose networks. Additionally, gene expression and metabolite level were measured in clones of trees grown in different environments. If these data were to be measured with the same samples collected at the same time, the correlations between gene expression and metabolite level could be used to enrich the current networks. In the future, we wish to take advantage of the active development of transcriptome and metabolome profile technologies to improve the quantification of gene transcripts and metabolites.

## Conclusion

Taken together, we used over 2.8 million SNPs primarily representing coding regions (exome) to perform associations with rarely investigated traits such as gene transcript abundance and metabolite levels in a range-wide association mapping population composed of unrelated genotypes. We identified a total of 1841 SNPs associated with 191 gene expression phenotypes and 524 SNPs associated with 53 metabolite level phenotypes. The identified SNPs reside in genes with a wide variety of functions. We constructed wood development and drought response gene networks and discovered key loci and genes that contribute to biological processes. This work provides candidate genes to study the genetic basis of gene expression and metabolism involved in complex biological processes. The identified genes and alleles are valuable resources for loblolly pine breeding through marker assisted selection and genomic selection. This work also highlights the efficiency of using association-mapping-based networks to discover key candidate genes involved in complex biological processes.

## Methods

### Plant material, genotypic data and phenotypic data

The loblolly pine association mapping population studied here was originally developed in the Allele Discovery of Economic Pine Traits 2 project (ADEPT2) [4, 33]. This population contains clones of progeny from parents selected from across the natural range from central Texas to Florida and north to Virginia. Functional gene transcripts and metabolite levels were measured in these trees as part of the ADEPT2 project [8–10]. Relative transcript abundance was measured using reverse transcription quantitative polymerase chain reaction (RT-qPCR) for 111 genes involved in xylem development and 88 genes involved in disease or drought response in woody tissue collected from 475 [8] and 354 [9] trees, respectively. Eckert et al. [10] measured the concentration of 292 metabolites in woody tissue collected from 297 trees, including 82 metabolites with known names.

In 2010, clones of the ADEPT2 population were established at the Harrison Experimental Forest of the Southern Institute of Forest Genetics near Saucier, Mississippi. We genotyped 375 trees in this population [14]. For the present study, the raw SNPs were filtered by accepting only bi-allelic sites with at least 5Χ sequencing coverage for all individuals without missing data and a minor allele frequency (MAF) ≥ 0.01. In total, 2,822,609 SNPs were retained for association analysis, and 94,478 haplotype blocks were detected in this population [15]. Genotyping data were only available for 212 trees with metabolite data and 278 trees with gene expression data. Therefore, 212 trees were used for association tests with concentration data for 82 metabolites and 278 trees were used for association tests with expression data for 199 genes. The gene expression phenotypes from the two data sets were organized into seven functional groups based on the biological processes in which they were involved: wood-related, disease-related, drought-related, reactive oxygen species (ROS)-related, terpenoid biosynthesis, programmed cell death (PCD), and phenylpropanoid pathway. The genes in each group were further assigned to sub-groups (Additional file 2: Table S6).

### Association analyses and annotation of genes that contained SNPs associated with phenotypes

The details of the association mapping analyses for the individual SNPs and phenotypes can be found at Lu et al. [15]. Briefly, the simple general linear model (GLM) method (*S* model) and the mixed linear model (MLM) method incorporating a kinship matrix (*K* model) and population structure covariate (*Q* model) were implemented by TASSEL 5.0 [34]. Because the populations from east and west of the Mississippi River displayed distinct population structures within this group [14], we named the trees from east of the Mississippi River (223 trees used for gene expression analysis and 184 trees used for metabolite concentration analysis) as the *east* population. Trees from west of the Mississippi River were not analyzed independently due to a low number of trees. The selectively neutral simple sequence repeat (SSR) markers were previously genotyped in this population [33]. Since these SSR markers were only available for 195 of the trees used for the gene expression analyses and 196 of the trees used for the metabolite concentration analysis, we named this group of trees the *str* population, and used them for a neutral population structure analysis. Therefore, three populations: *total* (*N* = 278), *east* (*N* = 223) and *str* (*N* = 195) populations, were used to perform association analyses for the gene expression data. Three populations, *total* (*N* = 212), *east* (*N* = 184) and *str* (*N* = 196), were used to perform association analyses for the metabolite concentration data. For the *total* and *east* populations, the *S* model and *K* model were applied. The kinship matrix was estimated using the SNP markers by TASSEL 5.0 [34]. For the *str* population, in addition to the *S* and *K* models, the *Q* model and the MLM incorporating both the kinship matrix and population structure covariate (*QK* model) were applied. The population structure covariate was estimated using the SSR markers and the software STRUCTURE [35, 36]. We determined the significant associations using a corrected Bonferroni threshold 0.05/94,478 = 5.29E-7, where 94,478 was the estimated number of haplotype blocks for this population.

The annotation for the genes containing the identified SNPs was obtained from loblolly pine genome annotation v3.0 (https://www.treegenesdb.org/FTP/Genomes/Pita/v1.01/annotation/) [37] or blastx alignment. The standalone blastx search was conducted using the default parameters and the best hits were kept. In the previous association studies, nearly 4000 EST-derived SNPs were associated with metabolite level and gene expression phenotypes [9, 10, 16]. To cross-reference associated SNPs identified in the current study with associated SNPs in the prior studies, we mapped the sequences with previously identified SNPs to loblolly pine reference assembly v1.01 (https://treegenesdb.org/FTP/Genomes/Pita/v1.01) [38] using the GMAP software [39]. The SNP-containing genes that encode transcription factors (TFs) were assigned to plant TF families according to the Plant Transcription Factor Database v4.0 (http://planttfdb.cbi.pku.edu.cn/index.php). The NCBI GI numbers of candidate genes were uploaded to the “Gene List Analysis” tool in the PANTHER Classification System (http://www.pantherdb.org) [40, 41]. The genes were mapped to the PANTHER databases and analyzed for their classification according to their molecular functions and protein classes.

The squared correlation coefficient (*R*^*2*^) between genotypes on the same scaffold was used as an LD measure and calculated using the “r2” function in the PLINK software [42]. We conducted *t*-tests to examine the significance of correlation coefficients between SNP pairs using the formula $\frac{r\sqrt{n-2}}{\sqrt{1-r2}}$. The resulting test statistic was used to calculate *P*-values. To correct for multiple testing, we applied the false discovery rate (FDR) method to adjust the original *P*-values at the α level of 0.05 [43]. To further inspect for haplotype blocks on these scaffolds, the squared correlation coefficient (*R*^*2*^) and adjusted *P*-values were presented in triangular heatmaps using the R package “LDheatmap” [44, 45].

### Network plots and SNP-SNP interaction analyses

To visualize the relationships between SNPs and their associated phenotypes, R package “igraph” was used to plot the networks [45, 46]. The network graphs were based on the functional groups we assigned. Blue, yellow and pink nodes represent SNPs, gene expression phenotypes and metabolite level phenotypes, respectively. Red and gray edges represent the significant SNP-metabolite-level and SNP-gene-expression associations. In addition, for the SNPs in the networks, the epistatic SNP-SNP interaction test was implemented using PLINK 1.9 [47]. The Bonferroni correction was applied to screen for significant SNP-SNP interactions. In the networks, purple edges represent the significant SNP-SNP interactions.

## Additional files

Additional file 1: **Table S1.** Annotation of genes containing SNPs associated with gene expression. **Table S2.** Annotation of genes containing SNPs associated with metabolite levels. **Table S3.** Expressed genes and numbers of SNPs associated with them. **Table S4.** Metabolites and numbers of SNPs associated with them. **Table S5.** Transcription factor (TF) genes containing SNPs associated with expressed genes and metabolites. (XLSX 1051 kb)

Additional file 2: **Table S6.** Main groups and subgroups of genes whose expression level associations with SNPs were analyzed in this study. **Table S7.** SNPs included in the wood development gene network. **Tables S8.** SNPs included in the drought response gene network. **Tables S9.** SNPs included in the ROS-related (a) and disease-related (b) gene networks. **Figure S1.** Distributions of *r*^2^ values for the SNP-gene expression (a) and the SNP-metabolite level (b) associations. **Figure S2.** Pairwise linkage disequilibrium (LD) values for SNPs in the scaffold 906 (a) and their *P*-values (b). **Figure S3.** Pairwise LD values for SNPs in the scaffold 897,738 (a) and their *P*-values (b). **Figure S4.** Pairwise LD values for SNPs in the scaffold tscaffold6003 (a) and their *P*-values (b). **Figure S5.** Pairwise LD values for SNPs in the scaffold tscaffold3539 (a) and their *P*-values (b). **Figure S6.** Pairwise LD values for SNPs in the scaffold tscaffold1180 (a) and their *P*-values (b). **Figure S7.** Pairwise LD values for SNPs in the scaffold tscaffold6112 (a) and their *P*-values (b). **Figure S8.** Pairwise LD values for SNPs in the scaffold tscaffold8336 (a) and their *P*-values (b). **Figure S9.** Pairwise LD values for SNPs in the scaffold tscaffold8193 (a) and their *P*-values (b). **Figure S10.** Pairwise LD values for SNPs in the scaffold tscaffold4407 (a) and their *P*-values (b). **Figure S11.** Gene networks comprised of SNPs significantly associated with expression of reactive oxygen species (ROS)-related (a) and disease-related (b) genes and metabolite levels. (PDF 2130 kb)

## Abbreviations

ADEPT2: Allele Discovery of Economic Pine Traits 2
CDS: Coding sequences
DREB: Dehydration responsive element binding protein
GA: Gibberellin
LD: Linkage disequilibrium
MAF: Minor allele frequency
P3’RS: Putative 3′ regulatory sequences
P5’RS: Putative 5′ regulatory sequences
PCD: Programmed cell death
ROS: Reactive oxygen species
SNP: Single nucleotide polymorphism
SSR: Simple sequence repeat
TF: Transcription factor
UTR: Untranslated sequences
