# Supplementary material for: Exploring the genetic basis of gene transcript abundance and metabolite levels in loblolly pine (Pinus taeda L.) using association mapping and network construction
Source: BMC Genet. 2018 Nov 6;19:100. doi: 10.1186/s12863-018-0687-7 (PMC6219081; doi:10.1186/s12863-018-0687-7)
Supplement: Supplementary file 2 — Table S6. Main groups and subgroups of genes whose expression level associations with SNPs were analyzed in this study. Table S7. SNPs included in the wood development gene network. Tables S8. SNPs included in the drought response gene network. Tables S9. SNPs included in the ROS-related (a) and disease-related (b) gene networks. Figure S1. Distributions of r2 values for the SNP-gene expression (a) and the SNP-metabolite level (b) associations. Figure S2. Pairwise linkage disequilibrium (LD) values for SNPs in the scaffold 906 (a) and their P-values (b). Figure S3. Pairwise LD values for SNPs in the scaffold 897,738 (a) and their P-values (b). Figure S4. Pairwise LD values for SNPs in the scaffold tscaffold6003 (a) and their P-values (b). Figure S5. Pairwise LD values for SNPs in the scaffold tscaffold3539 (a) and their P-values (b). Figure S6. Pairwise LD values for SNPs in the scaffold tscaffold1180 (a) and their P-values (b). Figure S7. Pairwise LD values for SNPs in the scaffold tscaffold6112 (a) and their P-values (b). Figure S8. Pairwise LD values for SNPs in the scaffold tscaffold8336 (a) and their P-values (b). Figure S9. Pairwise LD values for SNPs in the scaffold tscaffold8193 (a) and their P-values (b). Figure S10. Pairwise LD values for SNPs in the scaffold tscaffold4407 (a) and their P-values (b). Figure S11. Gene networks comprised of SNPs significantly associated with expression of reactive oxygen species (ROS)-related (a) and disease-related (b) genes and metabolite levels. (PDF 2130 kb) [file 12863_2018_687_MOESM2_ESM.pdf]

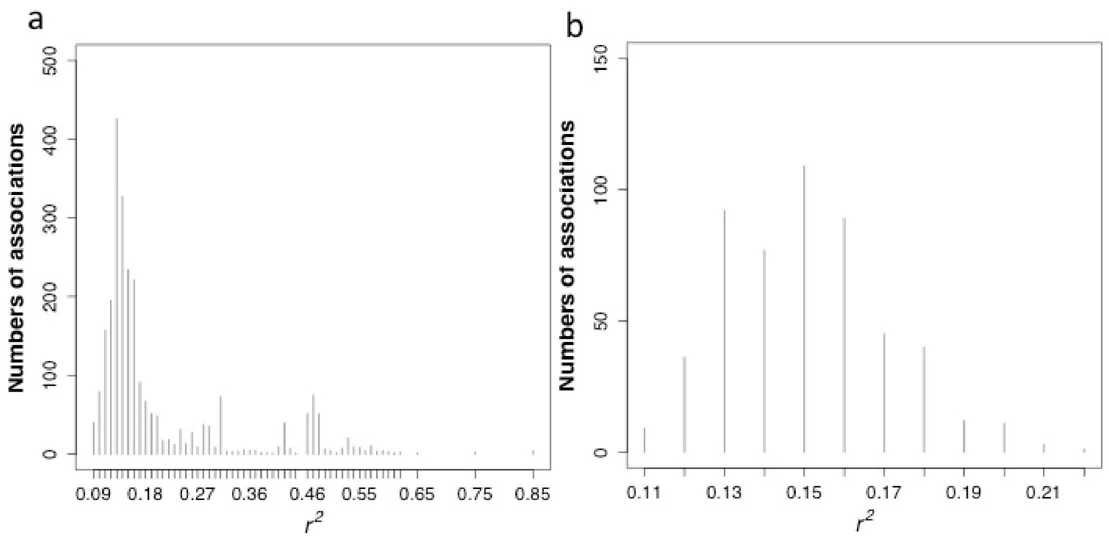

Fig. S1 Distributions of r^2 values for the SNP-gene-expression (a) and the SNP-metabolite-level (b) associations

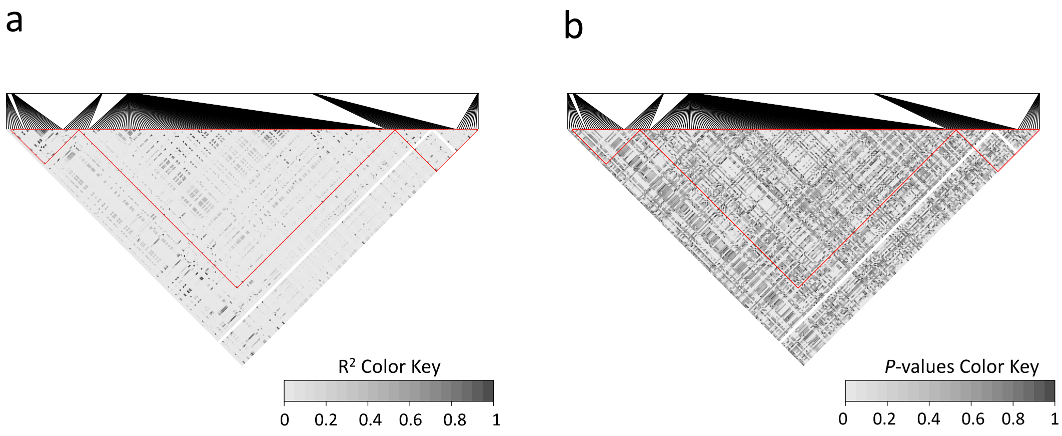

Fig. S2 Pairwise linkage disequilibrium (LD) values for SNPs in the scaffold 906 (a) and their *P*-values (b). The red line outlines LD values for SNPs tscaffold906_435061, tscaffold906_472382, tscaffold906_554605, and tscaffold906_619346. They were significantly associated with expression of the peroxidase gene *PtGPX3* ($r^2 > 0.24$). Significant pairwise LD values ($R^2 > 0.80$, $P < 0.01$) were detected between these four SNPs

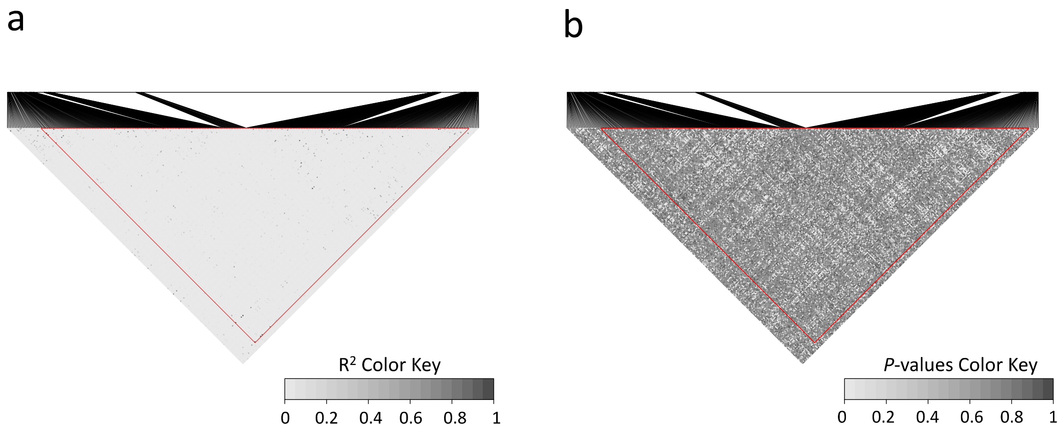

Fig. S3 Pairwise LD values for SNPs in the scaffold 897738 (a) and their *P*-values (b). The red line outlines LD values for two SNPs, scaffold897738_55448 and scaffold897738_68005. They were significantly associated with expression of the cellulose synthase like gene *CS-5828* (r^2 = 0.14). A significant pairwise LD value (R^2 = 1, $P < 0.01$) was detected between these two SNPs

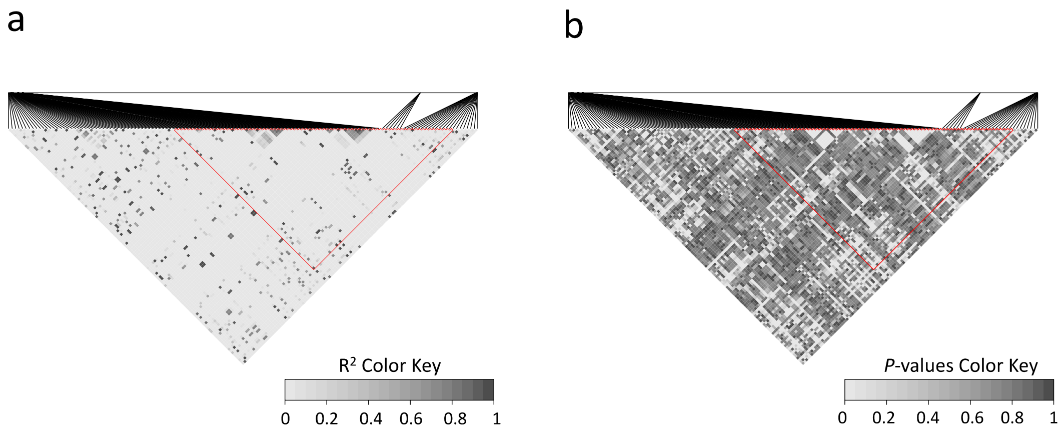

Fig. S4 Pairwise LD values for SNPs in the scaffold tscaffold6003 (a) and their *P*-values (b). The red line outlines LD values for two SNPs tscaffold6003_1005447 and tscaffold6003_763131. They were significantly associated with expression of the drought signaling related gene *ERD3* (r^2 = 0.13). A significant pairwise LD value (R^2 = 0.93, $P < 0.01$) was detected between these two SNPs

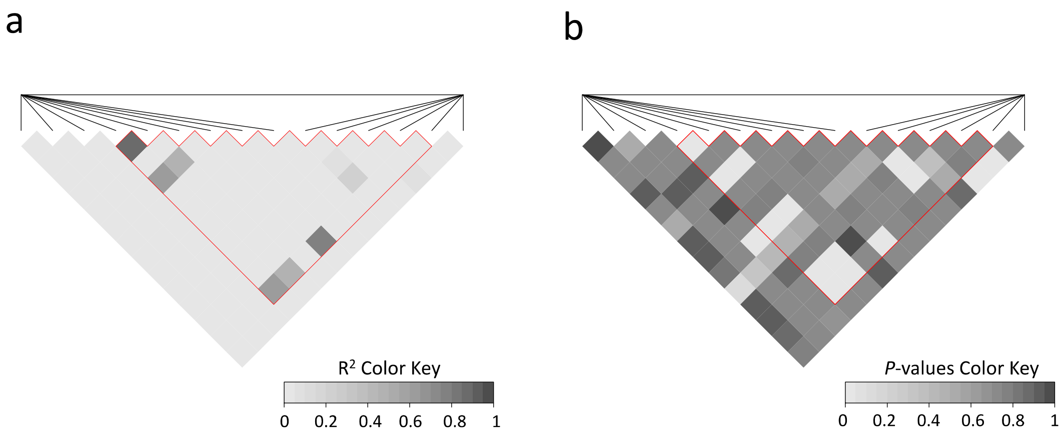

Fig. S5 Pairwise LD values for SNPs in the scaffold tscaffold3539 (a) and their *P*-values (b). The red line outlines LD values for two SNPs tscaffold3539_225325 and tscaffold3539_286987. They were significantly associated with expression of the drought-responsive transcription factor gene *HDZ32* (r^2 = 0.17). A significant pairwise LD value (R^2 = 0.6, P < 0.01) was detected between these two SNPs

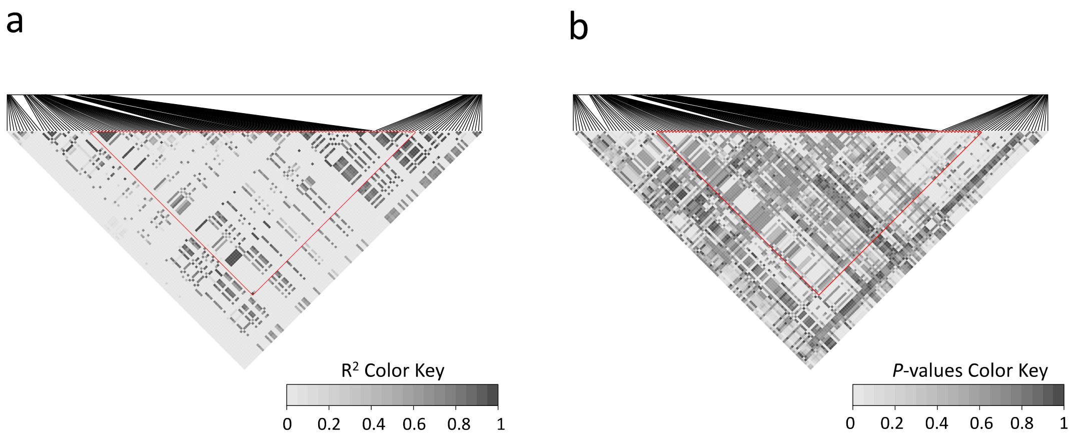

Fig. S6 Pairwise LD values for SNPs in the scaffold tscaffold1180 (a) and their *P*-values (b). The red line outlines LD values for two SNPs tscaffold1180_39656 and tscaffold1180_49757. They were significantly associated with expression of the wood development enzyme gene *prxC2* (r^2 = 0.13). A significant pairwise LD value (R^2 = 1, P < 0.01) was detected between these two SNPs

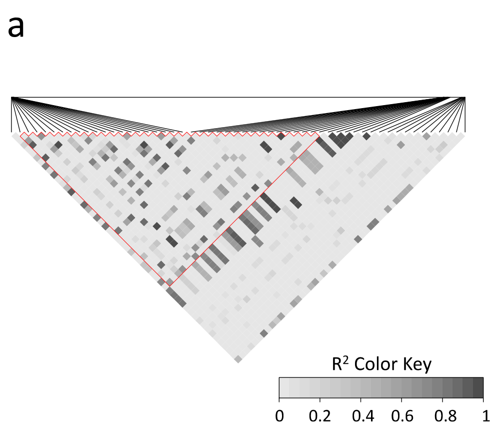

Fig. S7 Pairwise LD values for SNPs in the scaffold tscaffold6112 (a) and their *P*-values (b). The red line outlines LD values for two SNPs tscaffold6112_565501 and tscaffold6112_616079. They were significantly associated with expression of the late embryogenesis abundant protein gene *PtEMB4* (r^2 = 0.19 and r^2 = 0.18). A significant pairwise LD value (R^2 = 0.62, $P < 0.01$) was detected between these two SNPs

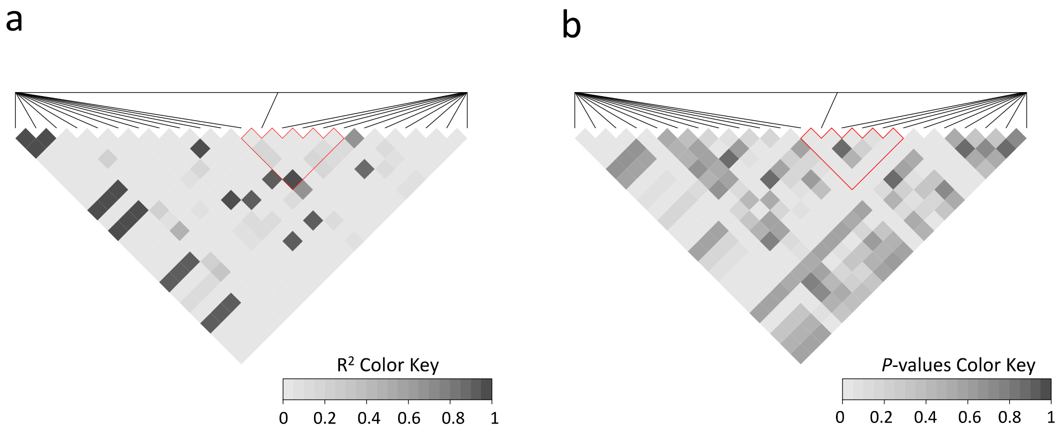

Fig. S8 Pairwise LD values for SNPs in the scaffold tscaffold8336 (a) and their *P*-values (b). The red line outlines LD values for two SNPs tscaffold8336_414590 and tscaffold8336_549532. They were significantly associated with expression of the detoxifying enzyme gene *PtGSTU18* (r^2 = 0.16). A significant pairwise LD value (R^2 = 0.98, $P < 0.01$) was detected between these two SNPs

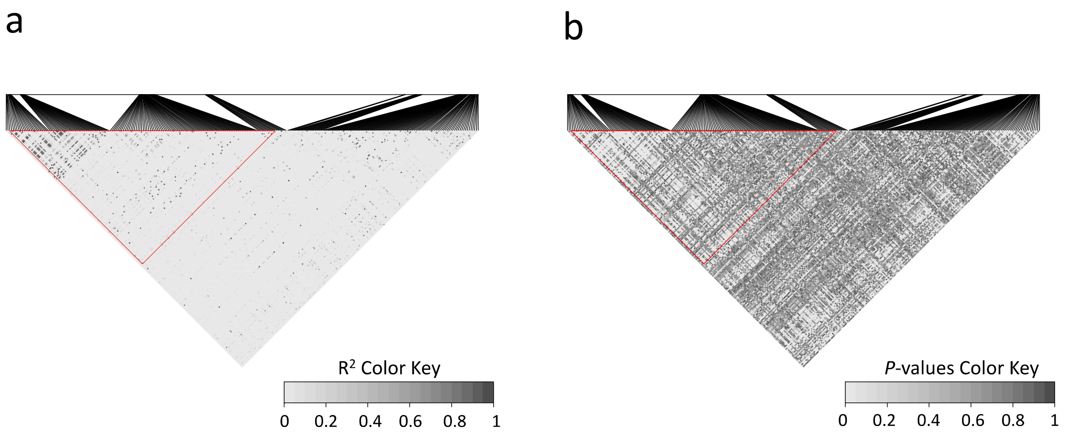

Fig. S9 Pairwise LD values for SNPs in the scaffold tscaffold8193 (a) and their *P*-values (b). The red line outlines LD values for two SNPs tscaffold8193_69949 and tscaffold8193_79785. They were significantly associated with expression of the drought signaling related *RPK1* gene (r^2 = 0.16 and r^2 = 0.13). A significant pairwise LD value (R^2 = 0.7, P < 0.01) was detected between these two SNPs

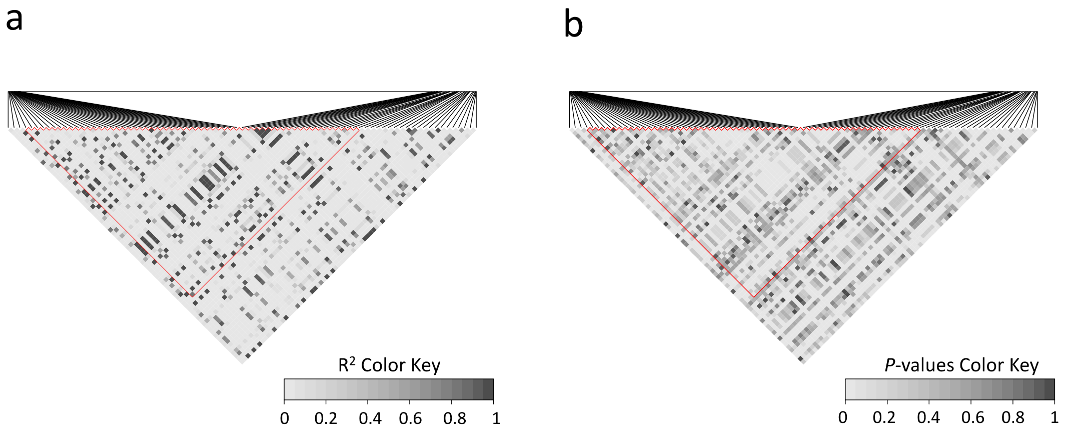

Fig. S10. Pairwise LD values for SNPs in the scaffold tscaffold4407 (a) and their *P*-values (b). The red line outlines LD values for two SNPs tscaffold4407_350690 and tscaffold4407_366097. They were significantly associated with expression of the wood development enzyme *gene SucSyn* (r^2 = 0.16 and r^2 = 0.12). A significant pairwise LD value (R^2 = 0.81, P < 0.01) was detected between these two SNPs

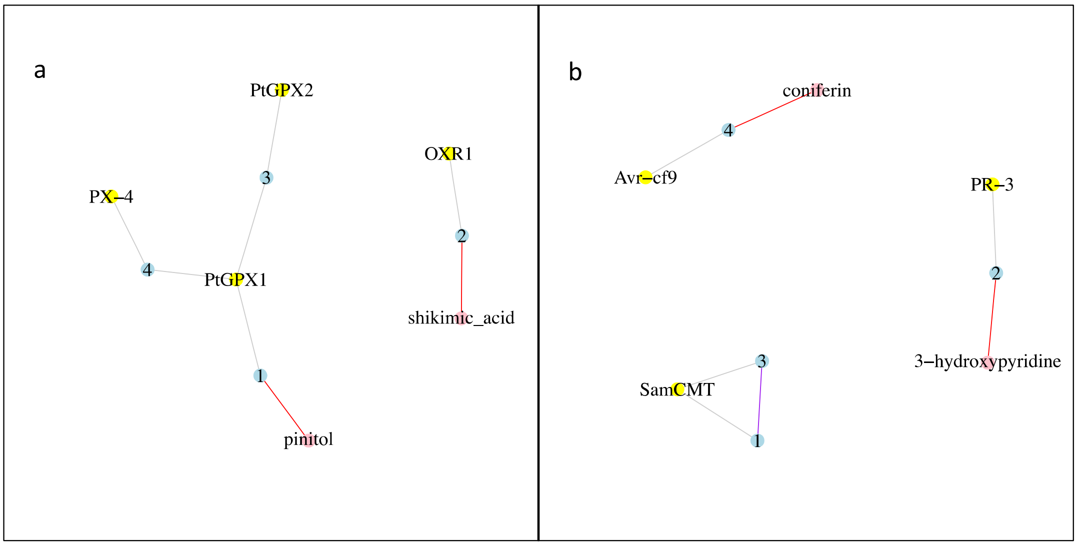

Fig. S11 Gene networks comprised of SNPs significantly associated with expression of reactive oxygen species (ROS)-related (a) and disease-related (b) genes and metabolite levels. The blue dot nodes represent SNPs. Details of the SNPs and the genes containing them are presented in Table S9. The yellow dot nodes represent genes, for which expression level was used as a phenotype trait in the SNP association analysis. The pink dot nodes represent metabolites, for which concentration level was used as a phenotype trait in the SNP association analysis. The grey and red edges represent SNP-gene-expression and SNP-metabolite-level associations, respectively. The purple edges represent significant SNP-SNP associations. *PX-4*, *PtGPX1*, and *PtGPX2* are peroxidase genes; *OXR1* is a ROS scavenging gene (a); *PR-3* is a pathogenesis-related gene, *Avr-cf9* and *SamCMT* are disease signaling genes (b)

Table S6 Main groups and subgroups of genes whose expression level associations with SNPs were analyzed in this study

Main groups	Subgroups
Wood-related	arabinogalactan-proteins; cell expansion; cell wall related; cellulose and callose synthase; cellulose synthase like; lignin biosynthesis enzyme; tubulin; wood development related; wood development transcription factor
Disease-related	disease signaling; disease-responsive transcription factor; pathogenesis-related protein
Drought-related	drought responsive; drought signaling; late embryogenesis abundant protein; osmotic adjustment
Reactive oxygen species (ROS)-related	detoxifying enzyme; H_2O_2 signaling; peroxidase
Terpenoid biosynthesis	terpenoid biosynthesis
Programmed cell death (PCD)	programmed cell death
Phenylpropanoid pathway	phenylpropanoid pathway

Table S7 SNPs included in the wood development gene network

#	SNP	Gene	Location	Gene function
1	C30981004_394	NA	NA	*BTB-POZ and MATH domain 6
2	C32254254_312	PITA_000082614	CDS	heat shock cognate 71 kDa protein
3	C32420248_14466	PITA_000072585	CDS	*dormancy/auxin associated family
4	C32510570_3431	NA	NA	*lipoxygenase LOX2
5	scaffold180470.2_16050	PITA_000089756	P3'RS	transmembrance protein, UPF0481
6	scaffold281669.2_43980	PITA_000066044	CDS	clathrin assembly protein, putative
7	scaffold292958_19955	PITA_000053202	intron	laccase 5
8	scaffold450207_158352	PITA_000021535	CDS	1-phosphatidylinositol 3-phosphate
9	scaffold450207_16949	PITA_000021533	P5'RS	pleiotropic drug resistance protein
10	scaffold450207_28939	PITA_000021533	CDS	pleiotropic drug resistance protein
11	scaffold610197_160281	PITA_000033627	CDS	cytochrome P450 71A1-like
12	scaffold668513_155213	PITA_000026911	CDS	D-xylose-proton symporter-like 2-like
13	scaffold778481_20006	PITA_000035225	P3'RS	pleiotropic drug resistance protein
14	scaffold786520_61917	PITA_000043838	CDS	putative chloroplast nucleoid DNA binding protein
15	scaffold806545_70099	PITA_000032971	CDS	*octicosapeptide/Phox/Bem1p domain
16	scaffold810906.2_4578	PITA_000054482	CDS	zinc finger A20 and AN1
17	scaffold833117_25452	PITA_000039745	CDS	probable arabinosyltransferase ARAD1
18	scaffold853861_77440	PITA_000050788	CDS	*disease resistance protein
19	scaffold859141_36865	NA	NA	*wall associated kinase 3
20	scaffold864246_4123	NA	NA	*U1 snRNP 70K truncated protein
21	scaffold898285_30519	PITA_000077607	CDS	calcium-dependent protein kinase
22	scaffold900291_50785	PITA_000070678	intron	pleiotropic drug resistance protein
23	scaffold901429.1_28954	PITA_000083498	CDS	probable LRR receptor-like
24	tscaffold1789_192012	PITA_000031787	CDS	histone H2A 11
25	tscaffold207_147293	PITAhm_001265	P3'RS	CBL-interacting protein kinase 04
26	tscaffold2073_179660	NA	NA	*cytochrome P450 CYP866A1
27	tscaffold2259_2500734	PITA_000000142	CDS	pentatricopeptide repeat-containing
28	tscaffold3181_438705	PITAhm_000620	P5'RS	sugar transport protein 7-like
29	tscaffold5561_114679	PITAhm_001053	CDS	uninformative
30	tscaffold5599_59726	PITA_000000691	CDS	E3 ubiquitin-protein ligase RHA1B-like
31	tscaffold6007_78709	PITAhm_000563	CDS	aspartokinase 3, chloroplastic-like
32	tscaffold7600_17911	PITA_000056578	P5'RS	uninformative
33	tscaffold823_284461	PITA_000011057	CDS	transcription factor GAMYB
34	scaffold129752_68497	PITA_000057129	CDS	putative peptide/nitrate transporter
35	scaffold344929_36100	PITA_000035657	CDS	eukaryotic translation initiation
36	scaffold40033.1_74895	PITA_000025698	CDS	60S ribosomal protein L8
37	scaffold500880.1_26348	PITA_000084405	CDS	disease resistance family protein / LRR family
38	scaffold500923.2_69645	PITA_000014956	CDS	uninformative
39	scaffold718601_15080	PITA_000091557	CDS	putative peptide/nitrate transporter
40	scaffold734383_27634	PITA_000076700	intron	D-xylose-proton symporter-like 3,

#	SNP	Gene	Location	Gene function
41	scaffold777716_52146	PITA_000030306	CDS	laccase-15-like
42	scaffold790940_46947	PITA_000031549	CDS	L-lactate dehydrogenase A isoform X2
43	scaffold814786_7638	PITA_000035012	CDS	chalcone synthase
44	scaffold884910_25465	PITA_000042265	5'UTR	serine/threonine-protein
45	scaffold904460_39241	PITA_000025946	CDS	not_annotated
46	tscaffold1826_327843	PITAhm_000605	intron	GTP pyrophosphokinase-like
47	tscaffold2183_148452	PITAhm_001264	intron	beta-galactosidase 8-like
48	tscaffold2632_76566	PITAhm_000592	intron	zinc finger protein, putative
49	tscaffold5883_187865	PITA_000026057	intron	glutaredoxin-C1
50	tscaffold739_104791	PITA_000028732	CDS	tir-nbs-lrr resistance protein
51	tscaffold7490_133973	PITA_000004476	CDS	tubulin-folding cofactor D-like
52	C32542874_98881	PITA_000049753	CDS	early endosome antigen 1

NA – not available (no information); *Based on the NCBI blastx results

Table S8 SNPs included in the drought response gene network

#	SNP	Gene	Location	Gene function
1	C17631075_89	NA	NA	*uninformative
2	C25503300_232	NA	NA	*pentatricopeptide repeat-containing protein
3	C27074060_197	NA	NA	*PQ-loop repeat family protein
4	C29987986_508	NA	NA	*protein sensitivity to red light reduced 1
5	C32101852_5207	PITA_000088922	CDS	wall-associated receptor kinase-like 14
6	C32126512_10138	PITA_000088025	intron	probable mediator of RNA polymerase II
7	C32206712_14378	PITA_000084933	intron	myrosinase-binding protein-like protein
8	C32225322_913	PITA_000084059	CDS	PHD finger protein alfin-like 5 isoform
9	C32385338_4467	PITA_000075456	CDS	GDSL esterase/lipase
10	C32511464_43552	PITA_000059768	intron	guanine nucleotide-binding protein-like
11	scaffold137825_3622	NA	NA	*uninformative
12	scaffold179894_28734	PITA_000074842	3’UTR	bidirectional sugar transporter
13	scaffold189278_295090	PITA_000019027	intron	heat stress transcription factor
14	scaffold195571.1_43779	NA	NA	*F6N18.1
15	scaffold195571.5_16807	NA	NA	*leukocyte immunoglobulin-like receptor family A
16	scaffold202109_286	PITA_000043702	CDS	pentatricopeptide repeat-containing
17	scaffold207198_20520	PITA_000041726	CDS	cellulose synthase A catalytic subunit 6
18	scaffold27614.1_18640	PITA_000068327	intron	probable acyl-activating enzyme 1,
19	scaffold297220_52495	PITA_000034468	CDS	cytochrome b245 beta chain homolog RbohAp108,
20	scaffold343479_46593	PITA_000020469	CDS	transcription factor bHLH120-like
21	scaffold406191.1_34526	PITA_000070639	CDS	heat shock 22K family protein
22	scaffold423393_11828	PITA_000071835	CDS	*phosphatidylethanolamine-binding protein
23	scaffold429437_57838	NA	NA	*villin 2, actin binding protein
24	scaffold452195_27866	PITA_000079940	CDS	DNA topoisomerase 2
25	scaffold46198.2_255696	NA	NA	*copia-like polyprotein
26	scaffold463075_199717	PITA_000018574	intron	*membrance trafficking family
27	scaffold473698_144131	PITA_000030131	CDS	lactosylceramide 4-alpha-galactosyltransferase
28	scaffold484562_126127	PITA_000036609	intron	galactomannan galactosyltransferase
29	scaffold536076_38366	PITA_000056077	P5'RS	pleiotropic drug resistance protein
30	scaffold63767_3786	PITA_000096272	P5'RS	actin
31	scaffold644480_1014	NA	NA	*CML42, calcium-binding protein
32	scaffold645183.2_8889	NA	NA	*SWEET1, bidirectional sugar transporter
33	scaffold654483_41318	PITAhm_001785	intron	uninformative
34	scaffold693997.1_29005	PITA_000055992	intron	GDSL esterase/lipase At1g74460-like
35	scaffold735351.1_13286	PITA_000089164	CDS	*glycosyltransferase,partial
36	scaffold7356_179786	PITA_000015934	CDS	oxidoreductase
37	scaffold738476_900	NA	NA	*armadillo/beta-catenin repeat family protein
38	scaffold767482_361	NA	NA	*sedoheptulose-bisphosphatase
39	scaffold798029.1_2133	NA	NA	*endonuclease/exonuclease/phosphatase family
40	scaffold821866_48362	PITA_000017703	P3'RS	not_annotated

#	SNP	Gene	Location	Gene function
41	scaffold832369_16631	PITA_000040812	CDS	phospholipase D beta 1-like
42	scaffold833746_241170	PITA_000018619	CDS	DNA helicase INO80-like
43	scaffold840945_91313	PITA_000051923	P5'RS	kinesin-4-like
44	scaffold844094.1_5034	NA	NA	*NBS/LRR, partial
45	scaffold844830.1_45638	PITA_000066120	CDS	LRR receptor-like
46	scaffold845535_2654	NA	NA	*uninformative
47	scaffold850532_11218	PITA_000081665	intron	L-ascorbate oxidase homolog
48	scaffold902827_38549	PITA_000057262	P5'RS	MLO protein homolog 1-like
49	tscaffold1547_1302045	PITA_000001347	CDS	prephenate dehydrogenase family protein
50	tscaffold5967_355315	NA	NA	*uninformative
51	tscaffold7243_134030	PITA_000036772	CDS	UDP-glucuronyltransferase-like protein
52	tscaffold7951_168276	PITAhm_002069	CDS	protein TRANSPARENT TESTA 12-like
53	C31677358_3957	NA	NA	*Cytochrome P450 CYP736B9
54	C31677358_4764	NA	NA	*Cytochrome P450 CYP736B9
55	C31816236_8140	NA	NA	*QUA1,nucleotide-diphospho-sugar transferases
56	C32267932_15399	PITA_000081970	CDS	cytochrome P450 71A1-like
57	C32382282_19212	PITA_000075685	5’UTR	transcription factor HY5-like
58	scaffold279240_125455	PITA_000032007	intron	expansin-B3 isoform X2
59	scaffold381187.1_77331	NA	NA	*transmembrane protein (DUF616)
60	scaffold456766_122010	PITA_000036264	intron	peroxygenase 2
61	scaffold506397_17855	PITA_000063949	P5'RS	14-3-3-like protein-like
62	scaffold576391_20949	PITA_000063006	CDS	L-type lectin-domain containing receptor kinase
63	scaffold592734.3_67301	PITA_000047923	CDS	L-type lectin-domain containing
64	scaffold69988_7043	NA	NA	*probable nucleoredoxin 1
65	scaffold798357_929	NA	NA	*sulfite oxidase
66	scaffold799261_13591	PITA_000091518	CDS	thioesterase/thiol ester dehydrase-isomerase
67	scaffold849303_43365	PITA_000041110	CDS	uninformative
68	scaffold877058_73274	PITA_000035202	CDS	chaperone protein ClpB1-like
69	scaffold880536_13279	PITA_000068161	P3'RS	60S ribosomal protein L7a
70	scaffold898125_21322	NA	NA	*myb domain protein 17
71	scaffold902635.2_44731	PITA_000059651	intron	homeobox-leucine zipper protein
72	tscaffold1060_405713	PITA_000003331	CDS	probable pectinesterase 53-like,
73	tscaffold5546_447295	NA	NA	*SUS2, sucrose synthase 2
74	tscaffold5995_12084	NA	NA	*caleosin-related family protein
75	tscaffold6642_165502	PITA_000023530	CDS	transaldolase 2
76	tscaffold7752_188476	PITAhm_002451	intron	calcium-dependent protein kinase 3-like
77	tscaffold7752_307810	PITAhm_002451	intron	calcium-dependent protein kinase 3-like
78	tscaffold823_284461	PITA_000011057	CDS	transcription factor GAMYB
79	tscaffold8401_260208	PITA_000019699	CDS	UPL2, similar to E3 ubiquitin protein ligase
80	tscaffold8494_634650	NA	NA	*uninformative

NA – not available (no information); *Based on the NCBI blastx results

Table S9 SNPs included in the ROS-related (a) and disease-related (b) gene networks

	#	SNP	Gene	Location	Gene function
a	1	C31147380_3098	NA	NA	*lipoxygenase family protein
a	2	scaffold170916_68414	PITA_000036089	CDS	putative leucine-rich repeat
a	3	scaffold4989_109854	PITA_000024055	CDS	probable polyadenylate-binding protein
a	4	tscaffold2536_712064	PITA_000006595	CDS	MATE efflux family protein
b	1	C17631075_89	NA	NA	*uninformative
b	2	scaffold28318.2_3363	NA	NA	*oligouridylate binding protein 1B
b	3	tscaffold1547_1302045	PITA_000001347	CDS	prephenate dehydrogenase family protein
b	4	tscaffold20_1281370	PITAhm_000077	intron	uninformative

NA – not available (no information); * Based on the NCBI blastx results
